# Capturing photochemical and photophysical transformations in iron complexes with ultrafast X-ray spectroscopy and scattering

**DOI:** 10.1039/d1sc01864g

**Published:** 2021-06-01

**Authors:** Kelly J. Gaffney

**Affiliations:** Stanford PULSE Institute, SLAC National Accelerator Laboratory, Stanford University Menlo Park California 94025 USA kgaffney@slac.stanford.edu

## Abstract

Light-driven chemical transformations provide a compelling approach to understanding chemical reactivity with the potential to use this understanding to advance solar energy and catalysis applications. Capturing the non-equilibrium trajectories of electronic excited states with precision, particularly for transition metal complexes, would provide a foundation for advancing both of these objectives. Of particular importance for 3d metal compounds is characterizing the population dynamics of charge-transfer (CT) and metal-centered (MC) electronic excited states and understanding how the inner coordination sphere structural dynamics mediate the interaction between these states. Recent advances in ultrafast X-ray laser science has enabled the electronic excited state dynamics in 3d metal complexes to be followed with unprecedented detail. This review will focus on simultaneous X-ray emission spectroscopy (XES) and X-ray solution scattering (XSS) studies of iron coordination and organometallic complexes. These simultaneous XES-XSS studies have provided detailed insight into the mechanism of light-induced spin crossover in iron coordination compounds, the interaction of CT and MC excited states in iron carbene photosensitizers, and the mechanism of Fe–S bond dissociation in cytochrome *c*.

## Abstract 2

Ultrafast X-ray scattering and spectroscopy captures photophysical and photochemical transformations of 3d transition metal complexes with atomistic detail.

## Graphical abstract

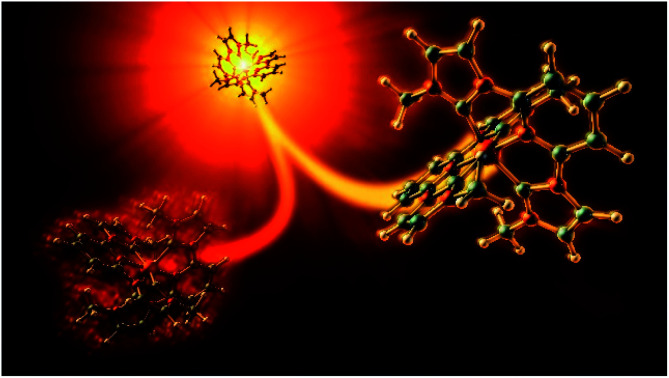

## I. Introduction

Energy transduction from sunlight to stable chemical fuels occurs through electronic excited states and highlights the need to characterize, understand, and ideally control, the electronic excited state properties of complex systems. The strong, non-adiabatic coupling of electrons and nuclei govern these excited state properties. This holds true for coordination and organometallic chemistry, where harnessing the strong optical absorption and photocatalytic activity of compounds depends on our ability to control fundamental physical and chemical phenomena governed by these dynamics.

While light initiated chemistry often occurs on time-scales extending from nanoseconds to milliseconds, the fate and functionality of light-generated electronic excited states is often determined by their evolution on the femtosecond to many picoseconds time scales. The initial charge separation in photosynthesis occurs in picoseconds,^1^ charge separation in organic and dye-sensitized solar cells often occurs in femtoseconds,^2,3^ and sub-picosecond photo-isomerization initiates chemical storage or signalling in various proteins from the rhodopsin family.^4,5^ The ultrafast evolution of electronic excited states can also inhibit function, such as the quenching of charge-transfer (CT) excited states in most 3d transition metal complexes by metal-centered (MC) excited states,^6–9^ in contrast to their isoelectronic 4d and 5d analogues^10–13^ and inhibit their utilization in photoredox chemistry. These exemplary cases clearly demonstrate the significant role ultrafast dynamics have on many light-driven chemical processes, presenting significant challenges for theory and experiment to characterize and understanding the evolution of electronic excited states.

**Fig. 1:**
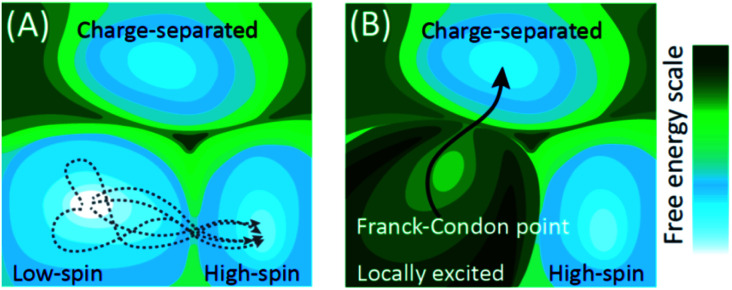
Schematic depiction of electronic ground and excited state potentials for a low-spin transition metal complex that also supports charge-separated (CS) and high-spin metal-centered (MC) excited states. (A) For the ground state potential energy surface (PES), the barrier to forming the high-spin MC excited state is lower than for the charge-separated state. The gray-dashed lines show the large range of trajectories launched from thermal equilibrium that can lead to the high-spin MC state formation. A prohibitively high barrier impedes formation of the change separated state. (B) Electronic excitation places the complex in a locally excited electronic state that can decay to either the high-spin MC or the CS state. The excited state PES and the initial conditions defined by the Franck–Condon point – the vertical projection of the ground state molecular geometry on the excited PES – direct the locally excited state towards the conical intersection with the CS state. This shows schematically how thermally inaccessible molecular configurations can be efficiently accessed with light-driven non-equilibrium dynamics.

Fig. 1 schematically shows how the conceptual framework for understanding the evolution of electronic excited states differs from that for electronic ground state transformations. Chemical transformations on both electronic excited or ground state potential energy surfaces (PES) involve changes in electronic states that accompany the changes in nuclear structure. However, the fundamental approximations that generally apply to ground electronic state chemistry more often than not fail to describe the dynamics of electronic excited states. As a general rule, electronic ground state transformations require crossing free energy barriers, where the rate of reactant equilibration greatly exceeds the rate of reaction. Under these circumstances, statistical models can be used to predict the probability of reaching the minimum barrier between reactant and product, the foundation of transition state theory.^14,15^ In the adiabatic approximation that underlies the generation of PES, degeneracies between PES exist, called conical intersections. The foundation of the adiabatic approximation is the separation of time scales for nuclear and electronic motion, but this separation fails at or near these conical intersections and necessitate the inclusion of the vibrational kinetic energy operator that leads to non-adiabatic transitions between electronic states.^16,17^ Unlike dynamics on the electronic ground state PES, photo-excitation creates distinct conditions where the initial position and momentum on the excited state potential surface can lead to chemical transformations governed by non-adiabatic interactions on the femtosecond timescale long before intramolecular vibrational energy redistribution. This scenario differs in essential ways from the conceptual framework for ground state reactivity because trajectories and non-equilibrium dynamics can and often are determinative and makes the statistical approximation that forms the foundation of transition state theory invalid for electronic excited states.

The conceptual framework for electronic excited state dynamics presented in Fig. 1 makes clear the challenge that faces both theory and experiment to advance the characterization and understanding of light-driven electronic excited state phenomena like light harvesting, photovoltaics, and photocatalysis. Explicitly, the non-equilibrium character of these excited state dynamics necessitates the generation of experimental and theoretical methods for following excited state trajectories with high fidelity. For theory this has focused on the development and implementation of quantum chemical molecular dynamics simulation methods. Time-dependent density functional theory has been the computationally most feasible approach to date,^18–20^ where the central challenge for transition metal complexes moving forward is finding electronic structure methods that capture the multi-configurational character of electronic excited states while being computationally tractable.^21^

The present limitations of theory and simulation for transition metal containing molecules and materials emphasize the importance of experiment in advancing our understanding. Specifically, experiment needs to incisively characterize the excited state trajectories to provide a foundation for a mechanistic understanding of excited state dynamics and a test-set for the development of new theoretical and computational methods. The established ultrafast optical methods have provided limited understanding for transition metal complexes for multiple reasons: optical methods do not provide easy access to the electronic spin dynamics that play a central role in transition metal systems, the inner coordination sphere vibrational dynamics occur in a spectral range difficult to access robustly with vibrational probes, and distinguishing between electronic and nuclear dynamics with electronic spectroscopy in the visible to UV range proves challenging.

The importance of electronic excited state dynamics in transition metal complexes and materials, coupled with the limitations of traditional ultrafast optical spectroscopy and current computational chemistry methods, has motivated the development of ultrafast hard X-ray spectroscopy and scattering to advance our mechanistic understanding of the dynamics in these systems. The developments have been multiple and benefited first from the efforts of many researchers at X-ray synchrotrons^22–37^ and more recently at X-ray free electron laser (XFEL) sources.^38–51^ In this review I will focus on simultaneous hard X-ray emission spectroscopy (XES) and X-ray solution scattering (XSS) measurements.^31,52–57^ This combination of experimental methods provides direct access to the charge and spin state of the metal center with XES and direct access to changes in metal–ligand bonding with XSS.^52,54–57^

**Fig. 2:**
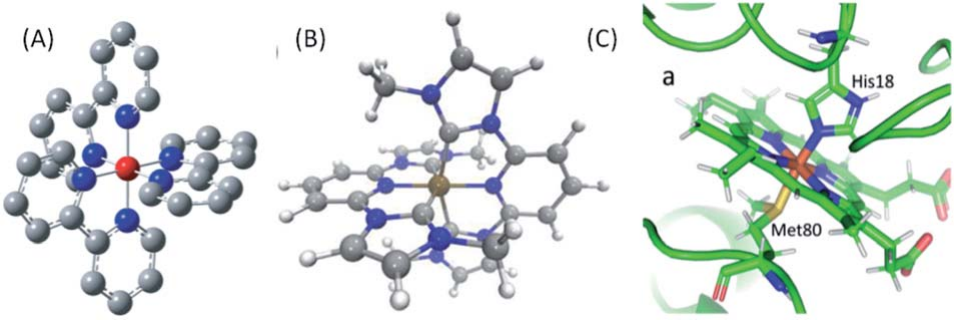
(A) [Fe(2,2′-bpy)_3_]^2+^, where bpy = 2,2′-bipyridine, (B) [Fe(bmip)_2_]^2+^, where bmip = 2,6-bis(3-methyl-imidazole-1-ylidine)-pyridine], (C) ferrous Fe(ii) heme of cytochrome *c*. (B) Adapted with permission from Pápai *et al.*^19^ copyright 2016 American Chemical Society. (C) Reproduced with permission from Reinhard *et al.*^57^ copyright 2021 Nature Research.

Femtosecond resolution simultaneous XES-XSS has been used to investigate the dynamics of photo-induced electron transfer in a Ru–Co molecular dyad and a mixed valence Ru–Fe complex,^52,56^ photo-induced spin crossover in [Fe(2,2′-bpy)_3_]^2+^, where bpy = 2,2′-bipyridine,^53,54^ photochemical dissociation of the Fe–S bond in cytochrome *c*,^57^ and the interaction between CT and MC electronic excited states in the iron carbene photosynthesizer, [Fe(bmip)_2_]^2+^, where bmip = 2,6-bis(3-methyl-imidazole-1-ylidine)-pyridine].^55^ This article will focus on the last three of these studies and the structure of all three chromophores can be found in Fig. 2.

This review will first discuss key attributes to the photophysics and photochemistry of 3d transition metal complexes. This will be followed by an assessment of XES and XSS, describing the strengths of the methods. The article will then present the mechanistic insight extracted from recent experimental investigations of [Fe(bpy)_3_]^2+^,^54^ cytochrome *c*,^57^ and [Fe(bmip)_2_]^2+^ (ref. 55) using the simultaneous XES-XSS method. The article will close with a discussion of future directions and a description of developments required to advance the understanding of the non-equilibrium dynamic of transition metal complexes and materials.

## II. Molecular degrees of freedom influencing 3d metal complex electronic excited states

A mechanistic understanding of electronic excited state dynamics requires following excited state trajectories and identifying the molecular geometries where transitions between electronic states occur with high probability. To achieve this objective requires clearly differentiating nuclear and electronic dynamics. While ultrafast electronic spectroscopies provide access to nuclear and electronic dynamics, changes in both generate similar spectral signatures. This makes interpretation of experimental observables challenging and often ambiguous. Ultrafast vibrational spectroscopy has advantages for assigning spectral features to distinct electronic and molecular configurations, but at the expensive of time resolution. The characteristic timescale for establishing a change in a vibrational spectrum depends intimately on the magnitude of the spectral shift and generally requires hundreds of femtoseconds to develop.^58^ This makes the initial electronic excited state evolution inaccessible to vibrational spectroscopy and limits the ability of ultrafast vibrational spectroscopy to characterize the dynamics of electronic excited states.

The strengths and weaknesses of ultrafast optical spectroscopy have left many important aspects of the non-equilibrium dynamics of transition metal complexes unresolved. For 3d transition metal complexes, the interplay of CT and MC excited states prove to be particularly significant. Generally, the lowest energy optically accessible electronic excited states of transition metal complexes have CT character. For the archetypical platinum group photocatalysts [Ru(bpy)_3_]^2+^ and [Ir(2,2′-phenylpyridine)_3_]^3+^, these CT states are the lowest energy electronic excited states, possess long excited state lifetimes, and efficiently initiate photoredox catalytic reactions.^59^ For 3d transition metal analogues of the platinum group complexes, such as [Fe(bpy)_3_]^2+^, the lowest energy optically accessible electronic excitation remains a CT state, but MC excited states involving changes in the spin and orbital occupancy of the 3d electrons often have lower energies and prove to efficiently quench CT excited states on the ultrafast time scale.^10^ This quenching of the CT states generally renders 3d complexes inefficient photosensitizers. This makes controlling the interplay between CT and MC excited states an essential component to the development of 3d photoredox catalysts and accentuates the central importance of directly accessing the molecular properties that most clearly differentiate CT and MC excited states.

**Fig. 3:**
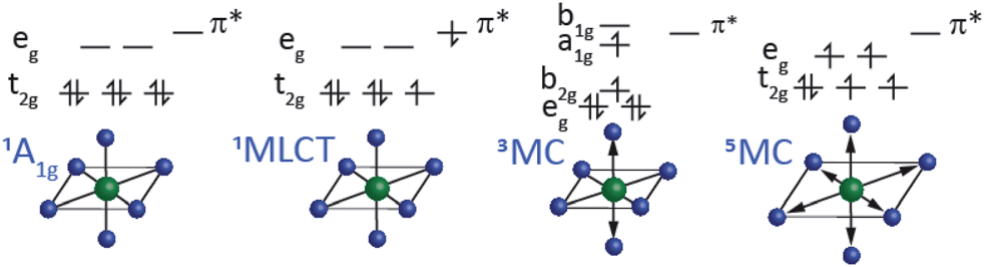
Schematic of the four electronic states most relevant to the electronic excited state dynamics for low-spin 3d^6^ ferrous complexes. The low spin ^1^A_1g_ configuration with symmetric octahedral bonding. The metal-to-ligand charge transfer (MLCT) excited state with a low spin 3d^5^ metal electronic configuration. Generally, the MLCT does not change the inner coordination sphere bonding significantly. The triplet metal-centered (^3^MC) excited state. This state has a single electron in the σ* metal–ligand anti-bonding e_g_ orbitals which leads to a tetragonal Jahn–Teller distortion (e_g_ converts to a_1g_ and b_1g_) and bond elongation. The high-spin quintet metal-centered (^5^MC) excited state restores the octahedral symmetry and the two electrons in the e_g_ orbital leads to greater bond elongation.

The most robust molecular signatures of MC and CT states depend on the number of d electrons, as well as the row of the periodic table for the metal, but given the important of six coordinate pseudo-octahedral *n*d^6^ transition metal complexes in photoredox catalysis applications,^59–61^ we will use the electronic and nuclear structure of *n*d^6^ complexes to highlight the molecular properties best able to characterize the interplay between the relevant electronic excited states. Fig. 3 schematically shows the properties of the four distinct electronic states of six coordinate pseudo-octahedral low spin 3d^6^ complexes, how they differ, and which experimental observables best differentiate the singlet ground state, ^1,3^MLCT, ^3^MC, and ^5^MC excited states. For these 3d^6^ complexes, the lowest energy optically accessible excited states have metal-to-ligand charge transfer (MLCT) character. This generates 3d^5^ configurations with an excited electron in a π* ligand orbital with either singlet or triplet character (^1,3^MLCT). As shown in Fig. 3, the MLCT excited states do not modify the metal–ligand bonding significantly. Ultrafast optical pump–probe and fluorescence spectroscopy provide robust signatures for MLCT excited states generally, with the intersystem crossing from ^1^MLCT to ^3^MLCT excited states being accessible with the decay of the fluorescence upconversion^62–64^ or the stimulated emission signal in a pump–probe measurement,^65^ and the ^3^MLCT excited states of many complexes can be identified by excited state absorption features associated with ligand radical anion transitions. The characteristics of these absorptions can often be assessed with spectroelectrochemistry measurements,^66,67^ particularly when interactions between the electron and the hole in the MLCT state only result in weak modification of the excited state absorption.

The challenge for ultrafast spectroscopy arises when the MLCT state relaxes to a MC state, because clear optical signatures of MC excited states have not been identified. This makes detailed characterization of the relaxation mechanism of CT excited states of 3d complexes largely inaccessible to ultrafast optical spectroscopy.

As shown in Fig. 3, ^3^MC excited states require the promotion of an electron from the t_2g_ orbital to the e_g_ orbital where the excitation also requires a change in electron spin moment. ^3^MC excited states for six coordinate 3d^6^ complexes have a single electron in the doubly degenerate e_g_ orbital. This drives Jahn–Teller symmetry reduction from octahedral to tetragonal symmetry and the splitting of the t_2g_ orbitals into e_g_ and b_2g_ orbitals and the e_g_ into a_1g_ and b_1g_ orbitals. This symmetry breaking also occurs for the metal ligand bonding, as shown in Fig. 3. Additionally, the e_g_ orbital has anti-bonding metal–ligand σ* character leading to an increase in mean metal–ligand bond length. For ^5^MC excited states, two t_2g_ electrons must be promoted to e_g_ orbitals where the orbital change for these two electrons also requires a change in spin state. This produces a larger, symmetric expansion of the metal–ligand bond lengths than for the ^3^MC excited state.

## III. Simultaneous XES-XSS studies of electronic excited state dynamics in 3d metal complexes

Fig. 3 shows schematically the critical observables for characterizing the electronic excited states of 3d^6^ octahedral complexes. We need experimental observables that clearly identify the metal-center spin moment and observables that clearly track changes in metal–ligand bonding. This review article will focus on the use of hard XES for the tracking of the metal spin moment of ferrous Fe(ii) complexes and the use of XSS to track changes in the metal–ligand inner coordination sphere bonding and will emphasize the essential mechanistic advantages of performing these measurements simultaneously to directly correlate electronic and nuclear dynamics in photo-excited 3d transition metal complexes.

### A. Ultrafast X-ray emission spectroscopy tracks charge and spin dynamics in 3d metal complexes

**Fig. 4:**
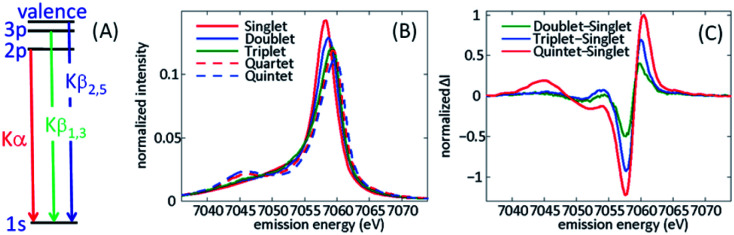
(A) Schematic of the X-ray emission process for iron following 1s core ionization. The Kα lines involve 2p filling of the 1s hole, Kβ_1,3_ lines 3p filling of the 1s hole, and Kβ_2,5_ lines involve valence electron filling of the 1s hole, often referred to as valence-to-core XES. (B) Spin state dependence of the Kβ_1,3_ spectra for a series of iron coordination complexes. (C) Difference spectra between the ferrous singlet state and electronic ground state doublet, triplet, and quintet states. (B and C) Adapted with permission from Zhang *et al.*^50^ copyright 2014 Nature Research.

Multiple spectroscopic observables are sensitive to the spin moment of transition metal complexes, such as Electron Paramagnetic Resonance (EPR) and Nuclear Magnetic Resonance (NMR), but the time-energy scale of EPR and NMR cannot access dynamics on the femto- to pico-second time scales. Transition metal core hole spectroscopies involving the core *n*p orbitals all have sensitivity to the electron spin moment at the metal site because of the strong angular momentum dependence of atomic spectroscopies.^68–70^ This review focuses on K-edge XES for 3d transition metals. K-edge XES involving *n*p filling of a 1s vacancy, such as 1s2p Kα and 1s3p Kβ XES,^70–75^ and shows sensitivity to the metal valence electron spin moment because of strong exchange coupling between the hole in the 2p or 3p level and the valence 3d spin in the final state generated by X-ray emission (Fig. 4(A)). The focus on K-edge XES reflects the particular properties of accelerator based XFEL. Single pass spontaneous amplification of stimulated emission (SASE) X-ray lasers have significant fluctuations in pulse spectrum that generally require the use of a monochromator to perform XAS measurements,^76^ though new approaches are being developed to eliminate the need for a monochromator.^77^ The monochromator effectively reduces the X-ray flux by two orders of magnitude and limits the signal quality, though multiple successful experiments have been performed to date.^43–46,51^ XES mitigates this limitation of SASE X-ray lasers because all the spectral information comes from the emitted X-rays, a property of the emitting atom not the X-ray laser pulse.

Fig. 4 has a schematic of XES, as well as characteristic spectra for Fe(ii) and Fe(iii) in different electronic spin configurations for Kβ XES. Our initial XES developments focused on Kβ XES because of the explicit sensitivity of the spectra to changes in spin shown in Fig. 4. In the analysis of our initial experimental studies, we assumed the electronic ground state of iron complexes with similar metal–ligand bonding would provide accurate model spectra for electronic excited states. This required the use of four-coordinate iron porphyrin and iron phthalocyanine complexes to model the intermediate triplet and quartet states, since octahedral d^5^ and d^6^ complexes cannot have intermediate spin electronic ground states. The use of ground state spectra for fitting the ultrafast dynamics of electronic excited states implicitly assumes the spectra do not depend strongly on changes in molecular geometry. Further development of Kα and Kβ XES theory and experiment have enabled a more nuanced and sophisticated use of XES and have also highlighted areas for continued development. The work of Serena DeBeer and colleagues warrants attention.^74,75^ As demonstrated by Pollock *et al.*, the Kβ XES spectra change with metal–ligand hybridization,^74^ often referred to as metal–ligand covalency. The contrast between sextet ferric [Fe(2,3,5,6-tetramethyl-benzenethiolate)_4_]^−^ and sextet FeF_3_ show the significant impact of the nephelauxetic effect, where the strong covalency of the Fe–thiolate bonds create a spectrum characteristic of a lower spin state due to delocalization of the spin density between the metal and ligands compared to the highly ionic FeF_3_. Continued developments also demonstrated the value of Kα XES, particularly for differentiating between singlet, doublet, and triplet spin configurations, where the Kα and Kβ spectra show similar shifts in the main line emission and the Kα cross section exceeds that for Kβ by roughly an order of magnitude.^71^ For Kβ XES, the position and magnitude of the lower energy satellite peak, creates clear advantages for the characterization of high spin configurations despite the lower emission cross section.^50,71^ The ESI associated with the article by Kunnus *et al.* compares the sensitivity of the Kα and Kβ XES to ligand variation for a series of strongly covalent low spin complexes, as well as sensitivity to changes in spin moment.^55^

**Fig. 5:**
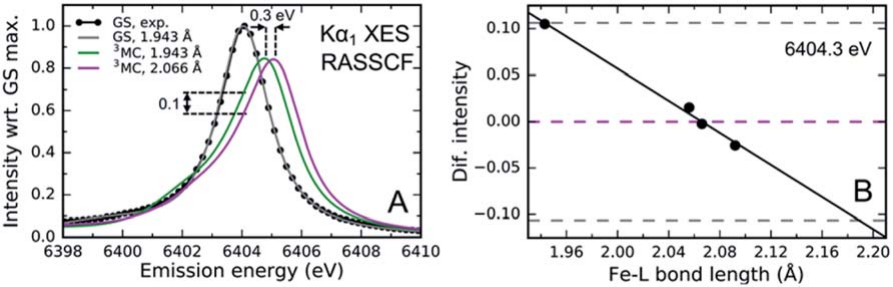
(A) Simulated and experimental Kα_1_ XES spectra for the singlet electronic ground state of [Fe(bmip)_2_]^2+^, as well as the simulated ^3^MC excited state spectra for two different mean metal–ligand bond lengths. (B) Calculated change in the ^3^MC state XES intensity at 6404.3 eV compared to the intensity at the optimal geometry as a function of the Fe–ligand (L) bond length (black dots; black line: linear fit). Dashed lines correspond to the intensity changes at optimal (magneta) and extremal (gray) bond lengths, *R* = 2.066 ± 0.123 Å. Figure adapted with permission from Kunnus *et al.*^55^ copyright 2020 Nature Research.

The sensitivity of the XES spectra to metal–ligand hybridization foreshadows the potential importance of metal–ligand bonding geometry, since nephelauxetic effects will be sensitive to changes in metal–ligand bond lengths for a given complex. As will be discussed in Section IV.B, we clearly observe oscillations in the Kα XES difference spectra for photoexcited [Fe(bmip)_2_]^2+^ that can be directly correlated with oscillations in the metal–ligand bond length.^55^ Here, I will focus on the theoretical explanation for these oscillations, as identified in the study by Vacher *et al.*^78^ As clearly shown in Fig. 5, the peak of the Kα emission line shifts with Fe–carbene bond length. The theoretical study of Vacher *et al.* clearly shows that the shift in the spectrum results from the different equilibrium metal–ligand bond length for the 1s and 2p core ionized electronic configuration of [Fe(bmip)_2_]^2+^ in a ^3^MC excited state.^78^ This vibronic effect should be general and occur for both Kα and Kβ XES, but the magnitude of the effect most likely depends on the metal ligand covalency and the specific emission line being measured. Addressing the importance of vibronic effects in K-edge XES requires further investigation.

### B. Ultrafast X-ray solution scattering tracks metal–ligand bonding dynamics in 3d metal complexes

The robust assessment of chemical dynamics and reactivity requires linking electronic and nuclear structure, as discussed in the introduction. Electronic spectroscopy accesses both electronic and nuclear dynamics, but only of Franck–Condon active modes for the probed electronic transition and the joint sensitivity adds significant complexity to the analysis. Ultrafast X-ray solution scattering (XSS) mitigates these issues by directly measuring changes in the nuclear arrangement. Michael Wulff, Hyotcherl Ihee, Martin Nielsen, and their collaborators and colleagues made essential contributions to the development of XSS with both synchrotron^31–37^ and ultrafast X-ray laser sources.^38–42^ This work provides a conceptual framework for thinking about the impact of optical excitation on a solute dissolved in a solvent. The difference scattering signal has three components: (1) changes in the scattering due to changes in the pair distribution function (PDF) for the solute, (2) changes in the solute–solvent PDF where the selection of atom pairs requires one atom to reside on a solute molecule and one on a solvent molecule, and (3) changes in the solvent PDF resulting from energy transfer from the solute.^32,36^Δ*S*(*Q*,*t*) = Δ*S*_solute_(*Q*,*t*) + Δ*S*_solvation cage_(*Q*,*t*) + Δ*S*_bulk solvent_(*Q*,*t*)

Extensive investigation has shown that component (3) can be effectively modelled with changes in the solvent structure factor resulting from changes in temperature for times scales before the macroscopic sample expansion that occurs on the nano- to microsecond time scale.^79^ These results indicate that the changes in the solvent scattering within the solvation shell around the solute does not differ sufficiently from the changes in the bulk solvent structure to be clearly observable experimentally. This allows the change in solvent structure due to energy transfer from the photoexcited solute on the sub-nanosecond timescale to be effectively modelled as a constant volume temperature change in the solvent structure factor.^79^

This leaves the chemically interesting components associated with changes in the (1) solute and the (2) solute–solvent PDF to be extracted from the XSS measurement. Component (2), where the PDF only sums over interatomic interferences where one atom resides on the solute and the other atom resides on the solvent, provides a unique perspective on solvation dynamics. Specifically, this aspect of XSS enables solvation dynamics to be followed with atomic site specificity and has the potential to provide a molecular perspective on solvation dynamics absent from the continuum descriptions of solvation that have largely been used to interpret ultrafast optical spectroscopy studies.^39,56^ This aspect of ultrafast XSS will not be reviewed in this article. Instead, the review will focus on the ability to directly track changes in the inner sphere metal–ligand bonding. Correlating these changes in bonding with changes in the transition metal charge and spin state provides a powerful foundation for mechanistic studies of electronic excited state dynamics.

Many challenges exist for extracting intramolecular structural dynamics from ultrafast XSS. For randomly arranged solutes in an isotropic solution, only information about the distances between atoms, the PDF, can be extracted from XSS,^80^ as made clear by the Debye formula,
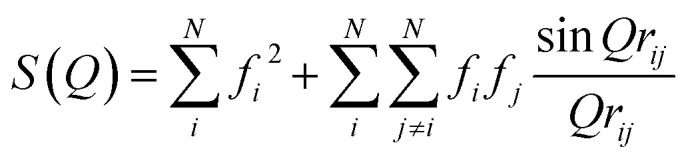
where *f*_*i*_ is the atomic form factor for atom *i* which scales with the total electrons per atom, *r*_*ij*_ is the distance between atoms *i* and *j*, and the momentum transfer
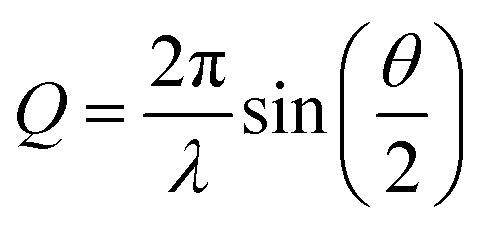
where *λ* is the X-ray wavelength and *θ* is the angle between the incident and scattered X-rays, and the sum is over all unique pairs of atoms in the sample. For increasingly complex molecules, the projection of the three dimension structure onto one experimental dimension significantly underdetermines the molecular structure without augmenting the analysis with additional knowledge and constraints. Multiple attributes of XSS have been used to address this challenge. Firstly, knowledge of the initial structure proves essential to tracking changes in the geometric structure. Secondly, the work I will be reviewing focuses on structural changes around the iron atoms in the molecules investigated because inner coordination sphere changes not only have significant mechanistic importance, but they also provide the strongest signals because the metal center in a transition metal complex has the largest *f*_*i*_ and usually dominates the scattering. Additional attributes of the measurement that can be used to advance the method will be discussed in the closing remarks.

## IV. Applying simultaneous XES-XSS to the photo-physics and photochemistry of iron complexes

### A. Mechanistic studies of light-driven spin crossover in iron transition metal complexes

Photo-induced spin crossover uses light to drive the transition from a low to a high spin electronic state. Our studies focused on understanding the photo-induced spin crossover mechanism as a model for understanding the quenching of CT states with MC excited states. Understanding this mechanism provides a potential pathway to long-lived CT excited states, a requirement for the development of 3d transition metal photocatalysts.

**Fig. 6:**
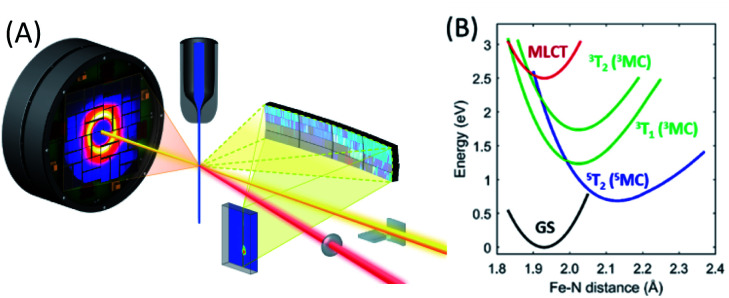
(A) Experimental schematic of the simultaneous ultrafast XES-XSS measurement. The crystal spectrometer at 90° from the incident X-ray beam (yellow) disperses the X-ray emission spectrum onto an area detector. The elastic XSS signal is collected in the forward direction onto a second area detector. An optical laser beam (red) photo-excites the sample delivered to the interaction region by a liquid jet. (B) The most relevant potential energy curves for photo-induced spin crossover in [Fe(bpy)_3_]^2+^ as a function of the Fe–N symmetric breathing mode coordinate calculated by Sousa *et al.*^82^ Figure adapted with permission from Kjaer *et al.*^54^ copyright 2019 The Royal Society of Chemistry.

Fig. 3 provides a schematic view of the electronic configurations relevant to photo-induced spin crossover, as well as characteristic changes in inner coordination shell bonding that accompanies transitions between these configurations. Light absorption excites a single electron, but spin crossover requires the excitation of two electrons, as well as spin flips for both active electrons. To convert between the low and high spin configurations following CT excitation requires electron transfer back to the metal, the internal conversion of a second electron, and the change in spin state for both of these electrons. The ultrafast X-ray spectroscopy measurements of McCusker, Shoenlein, and Huse, and those of Bressler and Chergui, established the sub-picosecond time scale of photoinduced spin crossover in 3d^6^ ferrous compounds,^23,81^ but the sequencing of the CT, internal conversion, and intersystem crossing steps, as well as the nuclear motions promoting these transitions remained largely unclear. These dynamic processes have universal importance for the understanding of the photophysics and photochemistry of 3d transition metal complexes, making photo-induced spin crossover an ideal case study for the development of simultaneous ultrafast XES and XSS shown schematically in Fig. 6(A).

Our work has focused on the archetypical photo-induced spin crossover complex [Fe(bpy)_3_]^2+^. Photo-excitation of the singlet ground state of [Fe(bpy)_3_]^2+^ generates a ^1^MLCT excited state that undergoes intersystem crossing to the ^3^MLCT state in 20 fs.^63^ McCusker and co-workers concluded from multiple optical pump–probe measurements on different polypyridyl Fe(ii) complexes that the ^5^MC state forms on the sub-picosecond timescale,^7,8,83^ but ultrafast UV spectroscopy^84^ and Fe L-edge X-ray spectroscopy probing of the unoccupied 3d orbitals^81^ provided the first direct observation of the ^5^MC electronic state being formed on the sub-picosecond timescale. The Fe L-edge spectroscopy measurement placed an upper bound of 200 fs on the spin crossover time constant.^81^ While indirection, ultrafast Fe K-edge X-ray spectroscopy measurements that primarily probe the inner coordination sphere bonding dynamics, provided direct evidence for the expansion of the Fe–N bond lengths that accompany the formation of the high-spin electronic configuration also occur with a time constant of less than 200 fs.^23^ These measurements provided definitive evidence for how fast spin crossover occurred, but did not provide a detailed mechanism.

Two particularly important aspects of photo-induced spin crossover remained unresolved. Firstly, does spin crossover involve the direct transition from the ^3^MLCT state to the ^5^MC excited state or does spin crossover occur sequentially, where the ^3^MCLT state first undergoes electron transfer to a ^3^MC excited state that then transitions to the ^5^MC excited state? Secondly, what vibrational coordinates promote the transitions between these excited states? Prior to simultaneous ultrafast XES-XSS studies of spin crossover, the answers to these questions remained in dispute. Chergui and colleagues used both Fe K-edge X-ray absorption and broad-band UV-visible probes to conclude the ^3^MLCT excited state transitions directly to the ^5^MC excited state.^23,85^ Both these measurements supported a continuous elongation of the metal–ligand bond, which supported a mechanism without a persistent ^3^MC intermediate. Lemke *et al.* used the improved time resolution and pulse intensity of XFELs to improve upon the pioneering measurement of Bressler *et al.* and observed coherent oscillations in the symmetric Fe–N bond length consistent with their continuous expansion.^44^ Lemke *et al.* did not exclude the potential importance of a ^3^MC transient in the spin crossover mechanism; they did conclude, however, that a kinetic model involving the exponential decay of a ^3^MC intermediate could not be rationalized with the observed Fe–N bond dynamics. Ultrafast XES probing of photo-induced spin crossover provided the strongest experimental evidence of a sequential mechanism involving a ^3^MC excited state transient.^50^ This presented a potential conflict between Fe–N bond dynamics extracted from the K-edge X-ray absorption measurements and the electronic state dynamics extracted from the XES measurements.

Theoretical calculations have also been instructive in the interpretation of experimental observations, but have not been decisive.^82,86–90^Fig. 6(B) presents the most relevant potential energy curves as a function of the Fe–N symmetric breathing mode coordinate from Sousa *et al.*^82^ Initial studies concluded that a sequential relaxation mechanism involving a ^3^MC should dominate,^82^ but more recent studies indicate direct ^3^MLCT relaxation to the ^5^MC excited state may compete with the sequential mechanism.^86,87^ The sensitivity of the second-order spin–orbit coupling magnitude to the specific geometry of [Fe(bpy)_3_]^2+^ in the ^3^MLCT excited state found by Sousa and co-workers^86,87^ demonstrates the need for direct dynamics simulations to theoretically determine the relative importance of the direct and sequential mechanisms.

**Fig. 7:**
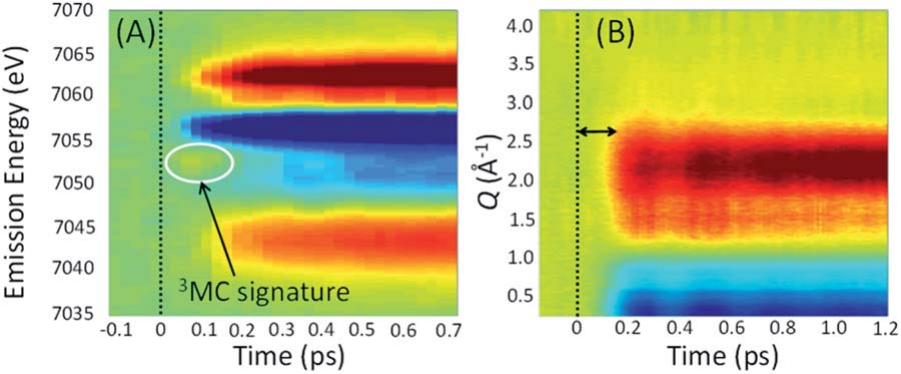
Simultaneous XES-XSS measurement on [Fe(bpy)_3_]^2+^ with time zero shown as a vertical dashed line. (A) Difference Kβ spectra with the ^3^MC excited state signature highlighted. (B) Difference XSS signal with the delay in the nuclear structural response emphasized. Additionally, the oscillations in the signal due to the Fe–N breathing mode vibrational wavepacket can be clearly seen.

Experimentally, the focus has turned to simultaneously measuring ultrafast XES and XSS and directly correlating changes in electronic state with changes in metal–ligand bonding. Haldrup *et al.* conducted proof of principle measurements on [Fe(bpy)_3_]^2+^ (ref. 53) and Canton *et al.* first demonstrated the technical feasibility of ultrafast simultaneous Kα XES-XSS measurements at an XFEL on a RuCo molecular dyad.^52^ By simultaneously measuring the ultrafast XES and XSS signals, as shown in Fig. 7, we correlated changes in electronic state with changes in molecular geometry. Qualitative inspection of the experiment lead to clear and important observations. The improvement in time resolution makes the signature of the ^3^MC excited state at 7054 eV shown in Fig. 7(A)^54^ clearer than the previous measurement.^50^ The change in nuclear structure observed with the XSS signal shown in Fig. 7(B) clearly follows the electronic excitation after a delay of roughly 100 fs and has a negative difference signal at low momentum transfer indicative of the molecular expansion that accompanies spin crossover. The appearance of oscillations in the XSS signal demonstrates spin crossover occurs sufficiently promptly to impulsively excite the low frequency Fe–N breathing mode, as initially assigned with ultrafast Fe K-edge absorption measurements^44^ and contrary to the assignment of the vibration to a bending mode in ultrafast optical measurements.^84^

A more quantitative analysis relied on a one-dimensional classical model for the vibrational dynamics of the metal–ligand symmetric breathing mode initially developed to explain the structural dynamics observed with ultrafast Fe K-edge absorption measurements.^44^ The direct correlation of the electronic and nuclear dynamics with the simultaneous XES-XSS measurement,^54^ buttressed by the ultrafast Fe K-edge absorption measurement,^44^ resolves the importance of the ^3^MC excited state in the spin crossover mechanism. Specifically, the XES measurement in isolation, did not warrant a more sophisticated analysis than a stepwise kinetic model.^50^ The simultaneous XES-XSS measurement enables explicit quantification of electronic and structural degrees of freedom.

By optimizing a model-description for the excited state relaxation on the multiple PES shown in Fig. 6(B) against the quantified electronic and structural parameters, it becomes possible to identify the dominant trajectory followed during electronic excited state relaxation and the relevant loci of intersections between electronic states from the experimental results.

**Fig. 8:**
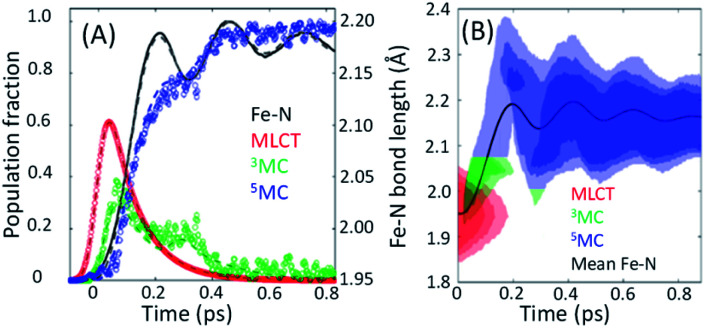
(A) The time dependent population dynamics of as a function of time for the MLCT, ^3^MC, and ^5^MC states, as well as the mean Fe–N symmetric bond length for the excited state population as a function of time. (B) The electronic excited state population and Fe–N bond length distributions consistent with the experimental results shown in (B). Figure adapted with permission from Kjaer *et al.*^54^ copyright 2019 The Royal Society of Chemistry.

The excited state populations and average Fe–N bond length dynamics of [Fe(bpy)_3_]^2+^ extracted from the XES-XSS data can be found in Fig. 8(A). As mentioned above, the improvement in XES data quality and time resolution demonstrate that the ^3^MC → ^5^MC transition is governed by non-exponential dynamics. This is readily visible from the plateau in the ^3^MC population at time delays between 200 fs and 350 fs, concurrent with a plateau in the ^5^MC population. The XES clearly shows a recurrence in the ^3^MC population coincident with the vibrational wave packet on the ^5^MC PES arriving at the inner turning point at roughly 350 fs. This demonstrates that the evolution of the ^3^MC state and the ^5^MC state populations formed from the MLCT relaxation depend directly on the underdamped oscillation along the Fe–N symmetric stretching coordinate. This leads to both forward and back transfer of population between the ^3^MC and ^5^MC states occurring over a narrow range of Fe–N bond lengths.

Within this one-dimensional Fe–N symmetric bond length description of motion on multiple PES, the molecules transitioning into the ^3^MC state from the MLCT state see a very steep gradient along the Fe–N bond length coordinate. The gradient generates a ballistic bond expansion bringing the system towards the intersection between the ^3^MC and ^5^MC excited states where transitions to the ^5^MC state occur with a very high probability. From the MLCT surface, each molecule has a time-independent transition probability to the ^3^MC surface, such that the MLCT ensemble decays with the 110 fs exponential lifetime identified in the X-ray absorption experiment.^44^ The systems are then propagated classically on the ^3^MC PES, until they reach a Fe–N distance defining the intersection point between the ^3^MC and ^5^MC surfaces. After reaching the outer turning point of the ^5^MC potential, the trajectories can revisit the ^5^MC–^3^MC intersection at which they have a probability of transitioning back to the ^3^MC potential. The simulation explicitly quantifies the excited electronic state and the Fe–N bond length of each molecule and reproduces the experimental observables from an ensemble average of simulated trajectories. Most significantly, the analysis can identify the Fe–N bond length where the ^3^MC → ^5^MC intersection occurs. Based on the PES calculated by Sousa *et al.*, the intersection between the ^3^MC and ^5^MC states occurs near the ^3^MC potential energy minimum at 2.05 ± 0.01 Å, whereas the subsequent ^5^MC → ^3^MC back transfer occurs very close to the calculated intersection for isotropic bond length changes at 1.98 ± 0.02 Å. The comparison between simulated dynamics for this model and the experimental dynamics appears in Fig. 8(A).

The different location for the intersection between the ^3^MC and ^5^MC states for the forward (^3^MC → ^5^MC) and back (^5^MC → ^3^MC) reactions could not occur if only one vibrational degree of freedom participated. This highlights the significance of multidimensional PES for understanding excited state dynamics.^91,92^ Most critically, the ^3^MC state will have a single electron in a 3d-dominated e_g_ orbital making the molecule pseudo-Jahn–Teller active (see schematic in Fig. 3). When molecules propagate on the ^3^MC PES, they will experience a tetragonal distortion gradient in addition to the Fe–N bond expansion gradient. This should be contrasted with the ^5^MC state which has a near octahedral coordination sphere with no tetragonal distortion. Consequently, the initial ^3^MC → ^5^MC transition should occur along a trajectory involving Fe–N bond expansion and tetragonal Fe–N bond distortion, while the ^5^MC → ^3^MC back transfer will involve trajectories dominated by oscillations in the Fe–N symmetric bond length. Ambiguity remains because we observe the projection of trajectories on the multi-dimension PES onto the symmetric Fe–N stretching coordinate; we do not directly resolve any other intramolecular structural degrees of freedom.

These ultrafast XES-XSS measurements have demonstrated the importance of ^3^MC states in the spin crossover mechanism of [Fe(bpy)_3_]^2+^, but the identity of the vibrational coordinates that promote the transition from the CT to the MC manifold of excited states remains unclear, but three observations from theory and experiment support an emerging picture: (1) the ultrafast measurements, in particular the ultrafast Fe K-edge XAS, provide strong evidence for the exponential decay of the ^3^MLCT excited state,^44^ (2) the UV-visible absorption spectrum, pump–probe signals associated with the ^3^MLCT excited state, and the potential energy curves calculated by Sousa *et al.* do not show a significant gradient on the ^3^MLCT PES (Fig. 6(B)),^44,54,63,82,84^ and (3) the calculated potential energy curves of Sousa *et al.* have the ^3^T_1_ and the ^5^T_2_ MC excited states crossing the ^3^MLCT near the minimum of the PES (Fig. 6(B)).^82^ All these observations support two conclusions. The decay of the ^3^MLCT state to the ^3^MC state depends on stochastic, short range motion on the ^3^MCLT potential where the magnitude of the coupling between the states strongly influences the lifetime analogous to barrierless non-adiabatic electronic transfer reactions.^93^

### B. Interplay between CT and MC excited states in iron carbene photosensitizers

The ligand field strength significantly influences the relative energy of charge transfer and metal-centered excited states, which in turn significantly influences the excited state dynamics. For the same ligand, the ligand field strength increases down a period for isoelectronic configurations as the 4d and 5d electrons extend further from the metal and increase the interaction with the ligand electronic structure.^6^ For example, this destabilizes the MC excited states of Ru(ii) and Os(ii) polypyridyl complexes when compared to their Fe(ii) analogues. For [Fe(bpy)_3_]^2+^, this makes ^3^MC and ^5^MC excited states lower in energy than the optically generated MLCT excited states, in contrast with [Ru(bpy)_3_]^2+^ and [Os(bpy)_3_]^2+^, and enabled the photo-induced spin crossover discussed in the previous section.

For applications benefiting from long-lived charge transfer excited states, like photovoltaics and photocatalysis, increasing the ligand field strength provides a clear path to suppressing the interaction between CT and MC excited states. Motivated by this objective, Wärnmark and co-workers focused on the synthesis of iron complexes with strong σ-donating N-heterocyclic carbene ligands for solar energy applications.^55,94–100^ The strong Fe–C σ-bonding in iron carbene complexes destabilizes the Fe e_g_ orbitals populated in MC excited states and increases their energy. Using the strong σ-bonding of carbene ligands has proven to be the most successful approach to date for extending the lifetime of CT excited states in iron complexes.^96,97,100^ The most impressive results have occurred for the ligand-to-metal charge transfer (LMCT) excited states of Fe(iii) carbene complexes like [Fe(phtmeimb)_2_]^+^, where phtmeimb is (phenyl[tris(3-methylimidazol-1-ylidene)]borate)^−^,^96,97,100^ which have nanosecond lifetimes and drive oxidative and reductive electron transfer reactions to methylviologen and diphenylamine.

Ferrous carbene complexes also show significantly longer MLCT excited state lifetimes than the archetypical ferrous polypyridyl complexes like [Fe(bpy)_3_]^2+^, though not as long as the LMCT lifetimes of the equivalent oxidized ferric carbenes.^94,100–102^ One such ferrous carbene photosynthesizer, [Fe(bmip)_2_]^2+^, where bmip = 2,6-bis(3-methyl-imidazole-1-ylidine)-pyridine] (see Fig. 2(B)), warrants attention because it shows very efficient electron injection when bound to titanium dioxide in a model dye sensitized solar cell.^103^ While transient absorption measurements had assigned a 9 ps lifetime to the MLCT excited state,^94^ quantum dynamics simulations indicate fast population of the ^3^MC excited states in [Fe(bmip)_2_]^2+^, with only 1/3 of the population remaining in the MLCT manifold and 2/3 forming a ^3^MC excited state with a ∼1 ps time constant.^19^ Clarifying the MLCT relaxation mechanism and addressing the discrepancies between the interpretation of ultrafast optical spectroscopy and quantum dynamics motivated our simultaneous ultrafast XES-XSS investigation of the potential role of ^3^MC excited states in the relaxation dynamics of the MLCT excited state of [Fe(bmip)_2_]^2+^ dissolved in acetonitrile.^55^

**Fig. 9:**
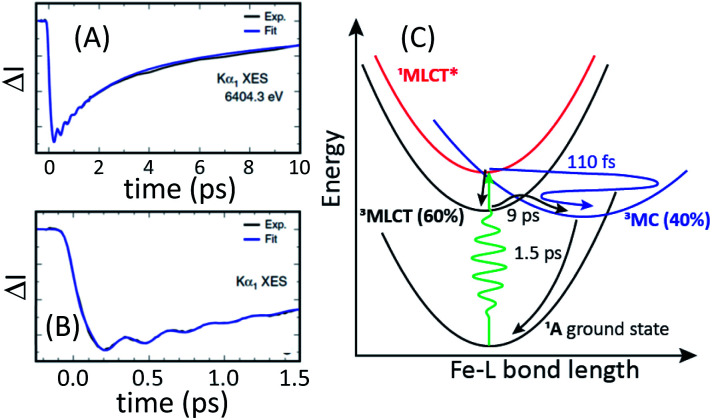
Time resolved difference intensity measured at the peak of the [Fe(bmip)_2_]^2+^ electronic ground state Kα_1_ spectrum for different temporal ranges (A and B). The bi-exponential decay in (A) and the delayed onset of the difference signal in (B) provide clear signatures of two relaxation pathways. A schematic of the relaxation mechanism can be found in (C). (A and B) Adapted with permission from Kunnus *et al.*^55^ copyright 2020 Nature Research.

The ultrafast XES-XSS experiment provides a detailed picture of coupled electronic and nuclear structural dynamics following MLCT excitation of [Fe(bmip)_2_]^2+^.^55^ As established with prior studies, the XES difference signal provides a powerful approach to tracking the population dynamics of both CT and MC excited states. Fig. 9 shows the time resolved Kα XES difference signal. Here we will focus on the electronic state population dynamics; the oscillations in the signal will be discussed below. The Kα difference signal, as well as the Kβ signal not shown, clearly reveal a delayed rise in the magnitude of the difference signal and a biexponential decay. This signal provides strong evidence for the relaxation of the initially excited MLCT* to both ^3^MLCT and ^3^MC excited states. Inspection of the Kβ XES difference spectra clearly shows the MC excited state has a ^3^MC, rather than ^5^MC, excited state character. We found that the MLCT* population bifurcates with 60% of the population relaxing to a ^3^MLCT state and 40% relaxing to a ^3^MC state *via* ultrafast back-electron transfer. This ^3^MC decays to the ground state with a 1.5 ps lifetime, while the ^3^MLCT state decays with a 9 ps lifetime, confirming the previously measured ^3^MLCT lifetime with UV-visible transient absorption experiments.^94^Fig. 9(C) summarizes the relaxation scheme consistent with the XES analysis.

The XSS signal accesses the intramolecular nuclear dynamics accompanying the electronic excited state relaxation. As demonstrated by Kunnus *et al.*,^55^ the large reduction in low-*Q* scattering provides the characteristic signature of metal ligand bond expansion, as discussed previously for [Fe(bpy)_3_]^2+^. Unfortunately, the measurement lacks the sensitivity to quantify the tetragonal distortion of the pseudo-octahedral coordination shell. The time-resolved XSS analysis for the ^3^MC excited state shows Fe–ligand bond expansion consistent with an average Fe–ligand bond length increase of Δ*R* = 0.123 Å predicted by theory.^104^ Since the electron transfer from MLCT* to the ^3^MC excited state occurs with a 110 fs time constant, this generates a vibrational wavepacket in the 278 fs period Fe–ligand stretching mode. The relaxation of the low-*Q* XSS signal associated with the Fe–ligand bond expansion decays with the lifetime of the ^3^MC state extracted from the analysis of the XES data.

As mentioned already, The Kα difference signal shows clear oscillations where the frequency and phase of the oscillation matches that seen in the XSS. This makes the vibronic origin of the oscillations in the spectrum clear. A brief discussion of this effect can be found in Section III.A. Here we will focus on a comparison of the results for [Fe(bmip)_2_]^2+^ and [Fe(bpy)_3_]^2+^. The ultrafast XSS results explicitly demonstrate the ultrafast population transfer from MLCT to MC excited state generates a metal–ligand bond breathing mode vibrational wavepacket. In the case of [Fe(bmip)_2_]^2+^, we have assigned the oscillations in the Kα difference spectra to changes in the nuclear structure, but in the case of [Fe(bpy)_3_]^2+^ we have assigned the modulation in the Kβ difference spectra for time delays in the 150 to 400 fs range to population transfer between the ^3^MC and ^5^MC excited states. The difference in interpretation reflects the fact that the XSS signal shows persistent oscillations for more than a picosecond delay for [Fe(bpy)_3_]^2+^, while the modulation in the Kβ XES spectrum only occurs when the vibrational wavepacket arrives at the inner turning point the first time. Stated concretely, if the modulation in the ^5^MC population shown in Fig. 8 at 300 fs was due to the vibrational wavepacket motion also shown in Fig. 9, the population would continue to oscillate at longer time delays, but we see no experimental evidence of such an effect.

We do not, however, conclude that the Kβ spectra for [Fe(bpy)_3_]^2+^ should have no sensitivity to molecular geometry. The origin of the geometry dependence of the Kα emission spectrum for [Fe(bmip)_2_]^2+^ discussed by Vacher *et al.* should also influence the Kβ spectrum.^78^ Specifically the optimal geometry of 3d coordination complexes should be different in the presence of a 1s core hole than it is for either a 2p or a 3p core hole. The expected generality of the mechanism does not require an equal sensitivity of Kα and Kβ emission spectroscopy to nuclear geometry and the sharper Kα emission lines may enhance the sensitivity to nuclear geometry.^78^ The sensitivity could also depend on the nature of the metal–ligand bonding. The work of DeBeer and coworkers has made clear the sensitivity of the XES spectra to covalency, where nephelauxetic effects lead to significant changes in the spectrum due to delocalization of the spin density onto the ligands.^74^ The strong σ-bond of Fe carbenes may accentuate the geometry dependence of the XES spectrum.

### C. Mechanistic studies of Fe–S bond photo-dissociation in cytochrome *c*

Understanding the mechanism of heme axial ligand photodissociation has been a long-standing challenge. Ultrafast ligand dissociation has been established by vibrational spectroscopies.^105–107^ For ferrous cytochrome *c* (cyt *c*), ultrafast resonance Raman identified a spectroscopic signature of five coordinate iron associated with the dissociation of the heme-Met80 Fe(ii)–S bond,^49,105^ but the electronic excited state that initiates the dissociation has not been clearly identified. Reliably detecting the short-lived electronic excited states involved in ligand photolysis of heme compounds with femtosecond optical pump–probe spectroscopy has proven ineffective.^108–110^ Light absorption generates a ^1^π–π* excitation of the porphyrin ring in heme proteins, an excitation that does not involve the electronic structure of the Fe(ii) site to any appreciable extent and does not directly lead to iron axial ligand dissociation. Photodissociation requires the population of an electronic state with a repulsive potential energy surface with respect to the iron axial bond. Some combination of electron transfer, internal conversion, and intersystem crossing is essential to the photodissociation mechanism, but the sequence of electronic states involved has not been identified conclusively.

For CO hemoglobin, ultrafast UV-visible pump–probe spectroscopy measurements have been interpreted by Franzen *et al.* to support ^1^π–π* state decay to a MLCT state.^111^ Generation of this MLCT state involves excitation of a d_π_(d_*xz*_,d_*yz*_) electron into the porphyrin π orbital vacated by light absorption. This weakens Fe–CO π back bonding and has been proposed to initiate Fe–CO dissociation. In the recent ultrafast XES study of NO photodissociation from myoglobin by Kinschel *et al.*, they found spectroscopy evidence for both CT and intermediate spin states on the sub-picosecond timescale when the Fe–NO bond dissociates, supporting the conclusion that bond dissociation precedes the formation of the high spin ^5^MC state.^112^

The MLCT mechanism proposed for Fe–CO in hemoglobin and Fe–NO in myoglobin should not be operative for Fe(ii)–S dissociation in cyt *c* because the Fe–S bond lacks π character.^113^ Chergui *et al.*, based on their ultrafast UV-visible pump–probe measurements, determined the electron in the π* orbital transfers to the metal d_z^2^_ orbital to initiate Fe(ii)–S dissociation.^108,109^ The d_z^2^_ orbital populated in this LMCT state has Fe–S antibonding σ* character and explains the driving force for bond dissociation, but prior studies indicate that the electron transfer from the π–π* to the LMCT state is energetically infeasible.^114,115^ Accordingly, the potential role of CT and intermediate-spin MC excited states involved in photodissociation of the Fe(ii)–S bond remained undetermined.

Our initial ultrafast X-ray spectroscopy study of cyt *c* used Fe K-edge XAS^45,46,116^ to confirm the photodissociation of the Fe–S bond and Kβ XES to confirm the five-coordinate Fe(ii) has a high spin quintet state structure, but did not identify the electronic excited state that initiated the Fe–S photodissociation.^49^ While the shape resonance in the Fe XAS spectrum does provide a signature for Fe–S photodissociation,^117^ as discussed in Section III.A, XAS requires a monochromatic X-ray beam and effectively reduces the flux by two-orders of magnitude. This makes measuring the XAS with sufficient time delays and energy point density challenging with current XFEL performance. This flux reduction also eliminates the ability to simultaneously measure the Kβ XES spectrum. Hence, our choice to use simultaneous XES-XSS for this mechanistic study of Fe(ii)–S bond dissociation.

**Fig. 10:**
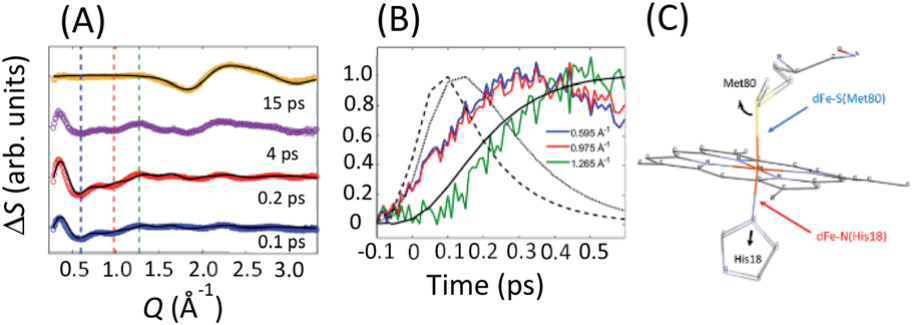
(A) XSS difference signal of cyt *c* for specific time delays. Black lines represent structural fits for the 0.1/0.2 ps curves and the fit of the 15 ps signal. The fits for 0.1/0.2 ps delays capture the changes in the axial coordination shown in (C). The 15 ps delay reflects the change in the water structure factor following the full recovery of the cyt *c* electronic ground state structure. (B) Time-dependence at *Q*-values indicated by dashed lines in (A). Black lines represent the ^1^π–π* (dashed), ^3^MC (dotted) and ^5^MC (solid) populations extracted from the Kβ XES. All curves are peak normalized for comparison. (C) Local structural changes are parameterized *via* Met80 rotation and His18 translation as illustrated by the black arrows. Figure adapted with permission from Reinhard *et al.*^57^ copyright 2021 Nature Research.

Using femtosecond resolution Kβ XES, we have identified a short-lived triplet metal-centered intermediate state with a 
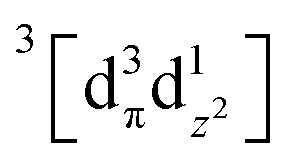
 (^3^MC) configuration. The Q-band ^1^π–π* excited state populates the ^3^MC state, which decays with an 87 fs lifetime to the Fe(ii) ^5^MC excited state with a 
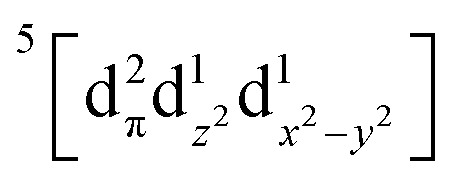
 (^5^MC) configuration. The normalized time dependence of the ^1^π–π*, ^3^MC, and ^5^MC excited state populations can be found in Fig. 10(B). While the ultrafast XES signal only robustly identifies the ^3^MC intermediate excited state, direct formation of this state from the π–π* excited state would require the concerted excitation of two electrons. Based on the previous studies of Franzen *et al.*^111^ and Falahati *et al.*,^118^ we propose the ^1^π–π* state decays *via* MLCT from the d_π_(d_*xz*_,d_*yz*_) into the porphyrin π hole with a 145 fs lifetime. MLCT state creation, and potentially intersystem crossing to the triplet excited state manifold, enables prompt LMCT from the porphyrin π* to the predominantly Fe d_z^2^_ orbital. These sequential charge transfers generate the ^3^MC excited state observed experimentally. The d_z^2^_ orbital populated in the ^3^MC has 
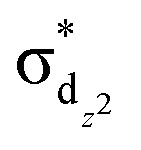
 dissociative character with respect to the Fe(ii)–S σ bond and initiates bond dissociation.

Fig. 10(A) shows the transient XSS signal in the *Q* = 0.2–3.3 Å^−1^ range and pump–probe time delays up to 600 fs.^33,49,119^ Our analysis focuses on the structural changes occurring during Fe(ii)–S bond dissociation. Fig. 10(A) clearly shows a prominent reduction in scattering between 0.4 and 1.1 Å^−1^ induced by photoexcitation that develops a characteristic shape and maximum amplitude faster than the rise in the quintet state population as shown in Fig. 10(B) and faster than the time for global protein structural changes.^119^ These observations support the assignment of the difference signal in this *Q*-range primarily to local structural changes associated with Fe axial coordination, which has informed our structural modelling of the signal.

We construct a model for the ultrafast nuclear dynamics focused on changes in the axial ligand positions. This model reflects the antibonding nature of the ^3^MC excited state with respect to the axial ligands, uses a minimal number of structural parameters to model the axial dynamics,^32,120^ and limits the analysis to the first 300 fs prior to global changes in the protein structure.^119^ Systematic modifications of these structural parameters clearly demonstrate that the negative difference signal seen between 0.4 and 1.1 Å^−1^ requires a ∼0.3 Å elongation of the Fe–Met80 bond and a ∼0.1 Å elongation of the Fe–His18 bond. Fig. 10(A) shows a comparison between fits of our model and the measured data at selected time delays and Fig. 10(C) shows the changes of the Fe–S(Met80) and Fe–N(His18) bonds consistent with the modelling of the XSS. Our model qualitatively reproduces the observed XSS difference signal of cyt *c* within the first 300 fs without Fe motion out of the heme plane (heme doming).^116,121^ This is consistent with the delayed appearance of the ^5^MC state that has been suggested as the primary origin of the doming motion due to the anti-bonding nature of the singly occupied d_x^2^−y^2^_ orbital with respect to the four Fe(ii)–N(porphyrin) bonds.^118,121,122^

**Fig. 11:**
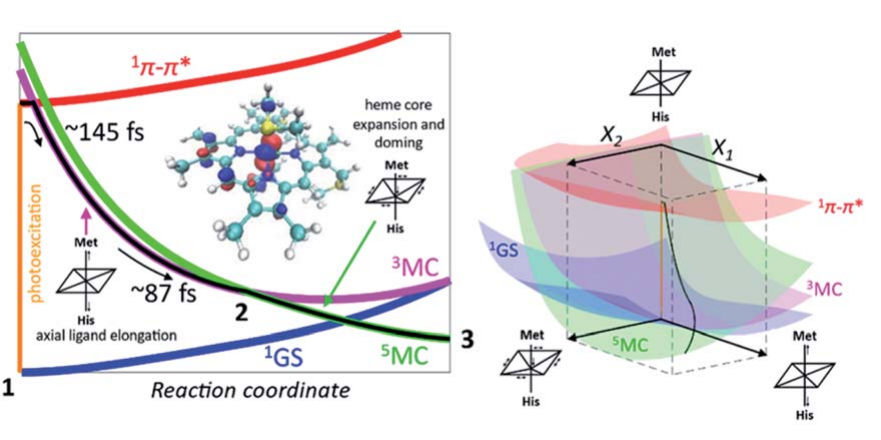
Mechanism for Fe–S bond photodissociation extracted from the simultaneous XES-XSS measurement on cyt *c*. The vertical orange line on the left represents the photoexcitation process and the black line is the proposed trajectory involving the ^1^π–π*, ^3^MC and ^5^MC states. The ^3^MC populates the σ* d_z^2^_ orbital and initiates Fe–S dissociation. The ^3^MC also creates a dπ hole that weakens the Fe–porphyrin π-bonding (inset, left). This leads to equatorial Fe–N (porphyrin) bond expansion and facilitates the crossing from the ^3^MC to the ^5^MC surface. The *X*_1_ and *X*_2_ coordinates in the 3D scheme on the right represents axial ligand elongation and heme core expansion and doming, respectively. A representative trajectory on the multiple potential energy surfaces is shown with a black line. Figure adapted with permission from Reinhard *et al.*^57^ copyright 2021 Nature Research.

Taken in total, the following mechanism for the photodissociation emerges. Fe–S bond dissociation requires the transition of the ^1^π–π* excitation to the Fe center. A sequence of two CT reactions, first a MLCT excited state and then a second CT to generate a ^3^MC state appears most likely, but our ultrafast XES measurements only confirms the formation of the ^3^MC state with a 145 fs time constant. Correlation of the electronic excited state population dynamics with the Fe(ii)–S bond elongation makes it clear that the dissociation proceeds the formation of the ^5^MC state. Fig. 11 provides a summary of the proposed photodissociation and recombination mechanism.

## V. Outlook

The past decade has seen a transformation of ultrafast hard X-ray science driven by X-ray free electron laser technology. While applications have been diverse, the impact of ultrafast hard X-ray science on chemistry has been dominated by studies of transition metal complexes. In parallel, significant advances in photoredox catalysis using the photophysical properties of transition metal complexes to drive molecular synthesis have occurred.^59–61^ These two advances provide significant opportunities to develop new photocatalysts from abundant metals and photocatalysts targeting specific chemical reactions.

The impact of ultrafast X-ray methods on chemistry has clearly transcended the value of demonstration experiments. For the three examples discussed in this review, mechanistic insights have been discovered for photo-induced spin crossover in iron coordination complexes, the role of metal-centered excited states in the excited state dynamics of iron carbene photosensitizers, and the Fe–S photodissociation mechanism in cytochrome *c*. The experimental methods discussed in this review should prove general and widely applicable to the electronic excited state dynamics of transition metal complexes.

Two aspirational goals warrant attention: replacing platinum group metals with abundant 3d transition metals in coordination and organometallic complexes and designing photocatalysts to target specific chemical reactions.^123–126^ XFEL measurements should make important contributions to advancing both of these objectives. Achieving these goals will require technical advances in theory, experiment, and synthesis and will also benefit from coordination and collaboration of scientists with diverse skills.

Focusing on ultrafast hard X-ray methods, the significant breakthroughs over the past decade should not obscure the important opportunities for further development. The significant shot-to-shot variation of XFEL sources make each shot an independent experiment and present significant challenges in learning how to knit tens of thousands of shots together into a robust experiment. The transition to superconducting electron accelerator technology will make XFEL performance more stable and enable shot averaging for reversible dynamics.^127,128^ The expansion of the photon energy range and repetition rate will also be significant. For X-ray solution scattering, the increased photon energy range will lead to needed improvements in spatial resolution and advances in X-ray pixel array detectors will continue to reduce the systematic non-linearity in detector performance. Additionally, the ultrafast time resolution of XFELs enables measurements to be performed before molecules rotate. The resultant anisotropy in the excited state XSS signal provides a two-dimensional view on the structural dynamics.^41,129–132^ Expanding the photon energy range will also allow ultrafast simultaneous XES-XSS measurements to be extended to 4d and 5d transition metal-complexes. This will be important for identifying how the photophysics and photochemistry varies for isoelectronic 3d, 4d, and 5d complexes. The increase in repetition rate will enable ultrafast X-ray spectroscopy based on weaker signals, including valence-to-core K-edge XES^133–135^ and extended X-ray absorption fine structure (EXAFS)^136^ measurements that directly characterize the inner coordination sphere geometry of the absorbing atom.

## Data availability

The data has already been published in prior publications since this is a review article. For this reason, I do not see the value of republishing the data.

## Author contributions

K. J. G. wrote this review article.

## Conflicts of interest

There are no conflicts to declare.
